# Role of Inflammatory Markers in Prognosis of Oral Squamous Cell Carcinoma

**DOI:** 10.31557/APJCP.2019.20.12.3635

**Published:** 2019

**Authors:** Thinali Sousa Dantas, Paulo Goberlânio de Barros Silva, Maria Elisa Quezado Lima Verde, Aloísio de Lima Ribeiro Júnior, Maria do Perpétuo Socorro Saldanha Cunha, Mário Rogério Lima Mota, Ana Paula Negreiros Nunes Alves, Renata Ferreira de Carvalho Leitão, Fabrício Bitu Sousa

**Affiliations:** 1 *Division of Oral Pathology, *; 3 *Department of Dentistry, *; 5 *Department of Morphology, Medicine, Federal University of Ceará, *; 2 *Department of Dentistry, Unichristus, *; 4 *Ceará School of Oncology, Hospital Haroldo Juaçaba, Fortaleza, Ceará, Brazil. *

**Keywords:** Inflammation, squamous cell carcinoma, survival, tumor necrosis factor, alpha

## Abstract

**Background:**

This estudie evaluated the immunostaining of cytokines in oral carcinoma, in tissue of margin of surgical resecate (MSR) and metastatic lymph nodes, as well as their role in patient prognosis.

**Methods:**

A retrospective study was carried out in patients with oral squamous cell carcinomas, and sociodemographic and clinical-pathological data were evaluated. In addition, surgical site analysis of the patients was conducted by immunohistochemistry, using a tissue microarray for inflammatory (Tumor Necrosis Factor-alpha, Interleukin-1beta, Interleukin-6, interleukin-10), transcription NF-kappa B and CD68 markers. Immunoexpression was assessed qualitatively and quantitatively using ImageJ software, and data were correlated with the prognostic factors and patient survival rates.

**Results:**

There was a greater immunoexpression of inflammatory and CD68 cytokines in primary tumour and lymph node metastasis than in MSR. In a multinomial logistic regression model, patients with low education (p = 0.041) and a high histoscore for TNF-α (p = 0.021) showed a survival rate of 15.64 (95% CI = 1.13-217.24) and 6.81 (95% CI = 1.02-105.96).

**Conclusion:**

Therefore, despite there is an increased immunoexpression of cytokines in the primary tumour, only TNF-α was the inflammatory cytokine that influenced the survival of patients with oral cancer.

## Introduction

Oral squamous cell carcinoma (OSCC) is most prevalent in the mouth and represents approximately 95% of mouth malignancies and is the 12 more prevalent in the world (Brasil, 2018).

 OSCC is the result of chronic exposure to carcinogens and evolves from a series of genetic and epigenetic alterations (Durazzo et al., 2005). In this context, inflammation has been shown to play an important role in the fields of malignization, tumour growth, local invasion, angiogenesis and metastasis. Although most of these mediators are produced to activate the immune system against the tumour, they can stimulate tumour growth (Balkwill and Mantovani, 2001).

 Tumour necrosis factor alpha (TNF-α) and numerous cytokines, such as interleukin 1 beta (IL-1β), 6 (IL-6) and 10 (IL-10), are associated with the activation of the cell cycle, leading to tumour progression and metastasis development (Eirón et al., 2012). The interaction between the stroma and the tumour parenchyma is essential for progression and can modify the prognosis by being a target for new approaches in cancer therapy (Dantas et al., 2016). The is more important in lymph nodes. In lymph nodes, the natural overproduction of cytokines can alter the behaviour of the neoplasia (Dantas et al., 2016).

Thus, the objective of this study is to evaluate the immunostaining of cytokines in OSCC, in tissue of margin of surgical resection (MSR) and metastatic lymph nodes and the role of these cytokines in patient prognosis and survival.

## Materials and Methods

This cross-sectional retrospective study evaluated 76 patients with OSCC according to the sample size calculation below. We selected patients with OSCC diagnosed and treated between the years 2011 to 2016 in the Hospital Haroldo Juaçaba. Data on clinical factors and socio-demographic was collect and the 5-year survival rate was calculated as previously described (Dantas et al., 2016).

*Sample Size Calculation*

Piva et al., (2013) showed that cases of OSCC that were the most poorly differentiated have a high expression of *TNF-α* in parenchyma cells (45.8%), compared with cases of OSCC that were well differentiated (18.2%). Based on the results of that study, we deemed it necessary to evaluate 66 patients in order to be able to reject the null hypothesis with 95% confidence and 90% power (Fleiss method with continuity correction). To account for the possibility of sample loss, the sample size was increased by 15%, bringing the total to 76 patients.

*TMA and immunohistochemistry*

The histologic slides were reviewed to select “host pots” with high cellularity, and we selected an area of the surgical site with a representative tumour microenvironment. For patients with lymph node metastasis, a representative tumour microenvironment in the sentinel lymph node was selected and a margin of surgical resecate (MSR) was too selected (Liu et al., 2016).

We used these areas (diameter: 2 mm) to make a TMA block using a Tissue Microarrayer (Quick-Ray UNITMA^®^). Then, microscopic slides (4 μm) were prepared conventional haematoxylin-eosin method and for immunohistochemistry.

After deparaffinization and rehydration, the tissue sections were submitted for immunohistochemical tecnique whit antigenic recovery was performed by heating in a citrate solution at pH 6.0, to peroxidase blocking with a 3% H_2_O_2_ solution diluted in Phosphate-Buffered Saline.

**Figure 1:**
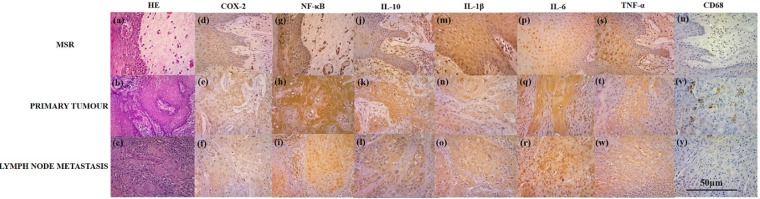
Histopathological Panel. Legend: Microscopic aspects of MSR (A), primary tumour (B) and lymph node metastasis (C) of patients diagnosed and treated at Haroldo Juaçaba Hospital, and histochemical profile for NF-κB (D, E and F), IL-10 (G, H and I), IL-1 (J, K and L), IL-6 (M, N and O), TNF-α (P, Q and R) and CD68 (S, T and W).

After protein blocking, we performed incubation overnight with the primary antibody CD68 (1:100, Abcam®, Cambridge, UK), TNF-α (1:100, Abcam^®^,), IL-1β (1:150, Abcam^®^,), IL-6 (1:300, Abcam^®^,), IL-10 (1:100, Abcam^®^,) and NF-ƙB (1:200, Abcam^®^) (Figure 1).

Universal Immuno-Peroxidase Polymer (Histoﬁne, Nicherei^®^, Tokyo, Japan) was utilized for the secondary antibody incubation. The visualization system used was 5,5 diaminobenzidine tetrahydrochloride (DAB) (Abcam^®^). In the negative control, the primary antibody was suppressed.

*Immunohistochemistry evaluation*

Five microscopic ﬁelds (400x) were photographed (Leica DM 2000^®^). The images were exported to ImageJ^®^ (cell counter command) to count the CD68 immunostained mononuclear cells and tumour cells exhibiting cytoplasmic or nuclear (for NK-kB) positivity for inflammatory markers. 

The percentage of positive tumour cells was categorized in scores ranging from 0 to 4 (0: no immune-positive cells; 1: 0-25% immune-positive cells; 2: 26-50% immune-positive cells; 3: 51-75% immune-positive cells; and 4: more than 75% immune-positive). Each case was also analysed for its intensity in scores ranging from 0 to 3 (0: no immune-positive cells; 1: mild immunostaining; 2: moderate immunostaining; and 3: intense immunostaining). The scores for the number and intensity of immunostaining were multiplied to obtain the histoscores (Range: 0-12) (Zhang et al., 2015).

*Statistical approach*

Data were analysed using statistical software SPSS (Statistical Package for Social Sciences) for Windows version 22.0, with a confidence level of 95% (P <0.05). The categorical data were analysed using a Chi-square test. The 5-year survival rate was also shown and analysed, and the mean and standard deviation were calculated and analysed using the Log-Rank Mantel-Cox test. The histoscores were shown as the standard deviation and compared using a Mann-Whitney or Kruskal-Wallis/Dunn test. A forward stepwise model was used to select variables that showed p<0.200 with the death rates for multinomial logistic regression. These results are shown as adjusted to hazard risk and a 95% confidence interval. 

*Ethical Correlations*

This study conformed to ethical principles and was accepted by the ethics committee of the Hospital Haroldo Juaçaba under protocol 1.552.674

## Results

*Clinical-pathological and socio-demographic data and 5-year survival of OSCC*

Of the 76 patients evaluated in this study, the majority were male (n=50, 65.8%), were more than 60 years old (n=43, 56.6%) and had tumours in the floor of mouth(n=35, 46.1%). The 5-year survival rate was 86.6% (n=66) and the survival mean was 49.11±25.13 months; these rates were not influenced by sex, age or localization of the tumour (Table 1).

T3/T4 tumours (n=47, 61.8%) and N0/1 tumours (n=50, 65.8%) were the most prevalent. Metastasis was shown in only 2 cases. T3/T4 tumours showed a lower survival rate (p=0.011) and a lower mean of survival (p=0.032). Lymph node metastasis and distant metastasis did not influence survival (Table 1).

The stage that was most prevalent was III/IV (n=60, 78.9%), the histologic gradation most prevalent was OSCC moderately differentiated (n=71, 93.4%), and the therapeutic approach most used was surgery and radiotherapy or surgery, radiotherapy and chemotherapy (n=32, 43.2%). The stage and histologic gradation did not influence survival, but the cases treated only with surgery showed superior survival compared to those subjected to combination treatments (p=0.010) (Table 1).

Of the 76 patients evaluated in this study, the majority of the patients were illiterate or had incomplete primary schooling (n=39, 51.3%), were mixed race (n=61, 80.3%), were from rural areas of the state (n=51, 67.1%), were married (n=55, 72.4%) and were smokers (n=30, 39.5%). Only education level significantly influenced survival; patients who were illiterate or who had incomplete primary schooling showed lower survival rates (p=0.014) (Table 1).

*Immunohistochemistry profile for inflammatory markers in OSCC, lymph node metastasis and MSR squamous epithelium and 5-years survival rates*

**Figure 2:**
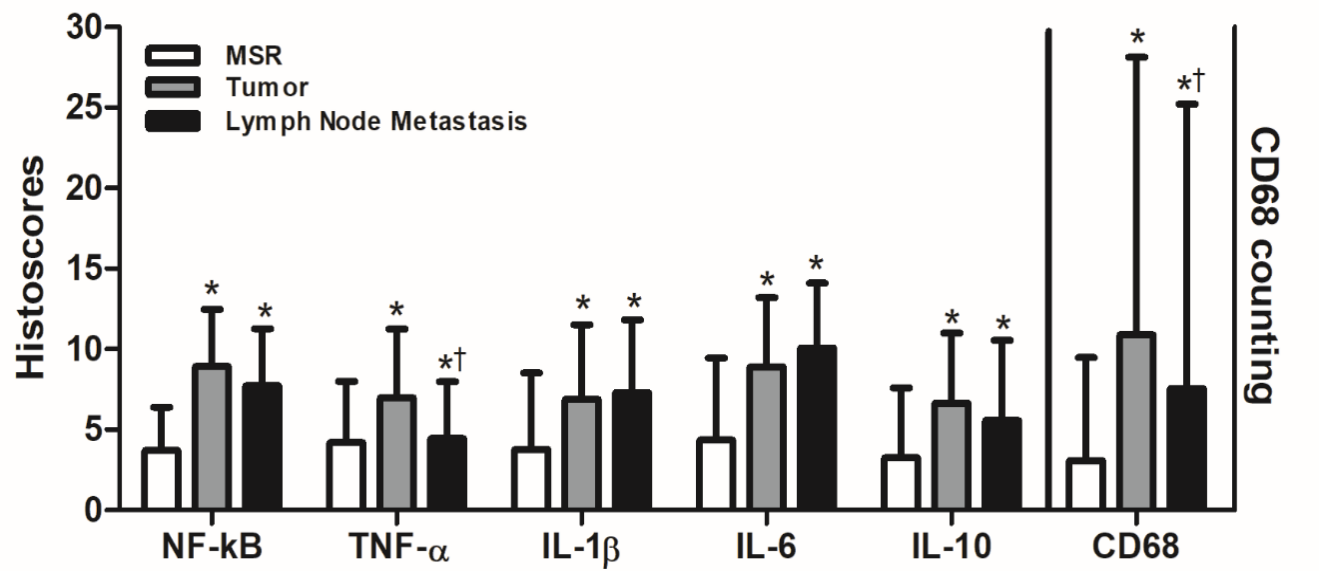
Immunoexpression Profile of Inflammatory Markers in Primary Tumours. MSR and metastasis in lymph nodes of SCC from the patients treated at Haroldo Juaçaba Hospital (Ceará Cancer Institute). Legend: *p<0.05 versus MSR. †p<0.05 versus Tumor; Kruskal-Wallis/Wilcoxon test (mean ± SD).

The CD68 mononuclear positive cells were significantly higher in tumour than in MSR squamous epithelium connective tissue and lymph node metastasis. The lymph node metastasis samples had significantly more CD68 mononuclear positive cells than did the connective tissue of MSR squamous epithelium (p<0.001) (Figure 2).

The NF-kB immunostaining was significantly higher in tumour and lymph node metastasis than in MSR squamous epithelium (p<0.001), and the TNF-α immunostaining was significantly higher in tumour than in lymph node metastasis and MSR squamous epithelium (p<0.001). IL-1β, IL-6 and IL-10 immunostaining was significantly higher in tumour and lymph node metastasis than in MSR squamous epithelium (p<0.001) (Figure 2).

NF-kB, IL-1β, IL-6 or IL-10 immunostaining and CD68 mononuclear positive cells in living patients did not differ in MSR squamous epithelium, tumour or lymph node metastasis when compared to that in deceased patients. However, deceased patients showed a higher TNF-α immunostaining in tumour (p=0.032) and lymph node metastasis (p=0.009) compared to living patients (Table 2).

*Multivariate analysis and interrelationship between prognostic variables of survival*

**Figure 3:**
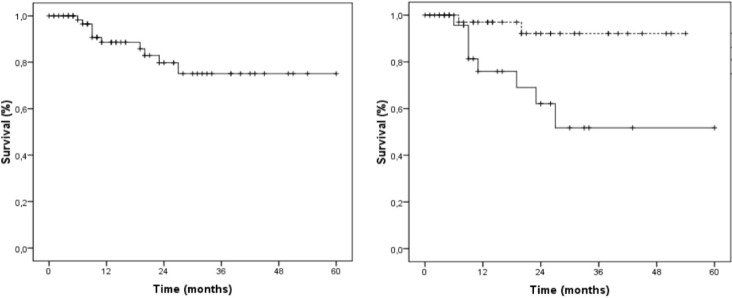
Survival Curve of Patients with SCC Treated at Haroldo Juaçaba Hospital (Ceará Cancer Institute). Legend: Patients with a TNF-α histoscore in a tumour superior to 6 and a low degree of education (Full line), as well as other groups (dashed line) (p = 0.003, Chi-square test; p = 0.005 Long-rank Mantel-Cox test).

The multinomial logistic regression model showed that patients with low education level (p=0.041, HR = 15.64, IC95% = 1.13 – 217.24) and high TNF-α immunostaining (p=0.021, HR = 6.81, IC95% = 1.02 – 105.96) had lower survival. When grouped, the patients with low education level and high TNF-α immunostaining showed lower survival rates (p=0.003, HR = 8.16, IC95% = 1.87 – 35.62) and lower mean survival (p=0.005) than other patients (Figure 3).

Lower TNF-α immunostaining in patients was not associated with stage and education level (p=1.000). However, in the higher TNF-α immunostaining patients, the III/IV stages were most prevalent in those with low education level (p=0.033, HR = 5.61, IC95% = 1.21 – 25.76). Additionally, the patients with higher TNF-α immunostaining and lower education levels were 3.82 (IC95% = 1.24 – 11.8) (Table 3) and and most were treated with surgery, radiotherapy and chemotherapy, rather than with surgery alone (p=0.026) (Table 4).

**Table 1:** Sociodemographic, Clinical-Pathological Profile and 5-Year Survival Follow-up of Patients Diagnosed with Oral Cancer at Hospital Haroldo Juaçaba (2011-2016).

	Sample		5-years survival			Survival time	
	n	%	n	%	p-valor^a^	(months)	p-valor^b^
Status Survival	66	86.8	-	-	-	49.11±25.13	-
Clinical and Pathological Profile							
Sex							
Male	50	65.8	41	82.00	0.15	39.45±21.37	0.14
Female	26	34.2	25	96.20		55.89±19.76	
Age							
Up to 60 years	43	56.6	36	83.70	0.499	42.28±25.33	0.356
Over 60 years	33	43.4	30	90.90		52.28±23.53	
Localization							
Floor of mouth	35	46.1	28	80.00	0.182	35.36±18.54	0.291
Thongue	26	34.2	25	96.20		49.00±23.60	
Others	15	19.7	13	86.70		52.35±18.80	
T							
T1/2	29	38.2	29	100.00	0.011	55.00±10.77	0.032
T3/4	47	61.8	37	78.70		44.94±27.47	
N							
N0/1	50	65.8	46	92.00	0.065	52.49±25.39	0.151
N2/3	26	34.2	20	76.90		37.60±21.11	
M							
M0	74	97.4	65	87.80	0.247	49.60±27.00	0.297
M1	2	2.6	1	50.00		23.50±14.50	
Stage							
1 and 2	16	21.1	16	100.00	0.08	52.49±14.37	0.125
3 and 4	60	78.9	50	83.30		46.92±27.85	
Histological Gradation							
Moderately differentiated	71	93.4	62	87.30	0.516	49.67±26.27	0.641
Poorly differentiated	5	6.6	4	80.00		23.00±10.00	
Treatment							
Surgery	10	13.5	10	100.00	0.037	56.10±6.64	0.074
Surgery + RT	32	43.2	30	93.80		55.38±17.66	
Surgery + RT + QT	32	43.2	24	75.00		36.48±23.37	
Sociodemographic Factors							
Education Level							
Illiterate/incomplete primary school	39	51.3	30	76.90	0.014	44.44±27.07	0.062
Completed primary school/High school/Higher education	37	48.7	36	97.30		51.76±13.29	
Convenat							
Plubic Health System	73	96.1	63	86.30	1	48.11±28.80	1
Private Health System	3	3.9	3	100.00		48.00±7.21	
Race							
Mixed	61	80.3	53	86.90	1	42.14±21.78	0.68
No-Mixed	15	19.7	13	86.70		50.36±23.36	
Origin							
Metropolitan Region	25	32.9	21	84.00	0.608	47.40±26.93	0.752
Countryside	51	67.1	45	88.20		43.40±20.15	
Marital Status							
With marriage bond	55	72.4	49	89.10	0.449	45.29±23.96	0.47
Without marriage bond	21	27.6	17	81.00		46.29±26.89	
Habits							
Smokes	30	39.5	28	93.30	0.341	45.40±16.79	0.483
Smokes/drink	21	27.6	18	85.70		44.75±22.01	
Don’t Smoke/drink	25	32.9	20	80.00		44.42±28.75	

**Table 2:** Influence of the Immunoexpression Profile of Inflammatory Markers on Primary Tumour. perilesion and lymph node metastasis in the rate of dead in patients with SCC of the mouth treated at Haroldo Juaçaba Hospital (Ceará Cancer Institute).

	Alive	Dead	p-Valor
CD68 counting			
MSR	3.26±6.81	1.88±3.23	0.642
Tumor	10.90±16.95	10.80±19.80	0.676
Lymph Node Metastasis	4.90±12.22	15.43±28.45	0.346
Histoscores			
NF-kB			
MSR	3.81±2.82	2.88±1.36	0.731
Tumor	9.11±3.44	7.70±4.16	0.275
Lymph Node Metastasis	7.67±3.66	8.00±3.27	0.89
TNF-α			
MSR	4.21±3.93	4.10±3.14	0.801
Tumor	6.55±4.21	9.60±3.86*	0.032
Lymph Node Metastasis	3.78±3.19	9.00±2.00*	0.009
IL-1β			
MSR	3.76±4.72	3.78±5.14	0.948
Tumor	6.56±4.61	8.80±4.54	0.438
Lymph Node Metastasis	7.00±4.33	9.00±6.00	0.409
IL-6			
MSR	4.23±5.20	5.00±4.55	0.691
Tumor	8.86±4.34	9.00±4.35	0.95
Lymph Node Metastasis	9.63±4.33	12.00±.00	0.235
IL-10			
MSR	3.43±4.32	2.10±4.48	0.356
Tumor	6.74±4.45	5.90±3.93	0.628
Lymph Node Metastasis	5.86±4.91	3.50±5.69	0.281

*p<0.05. Mann-Whitney test (mean ± SD).

**Table 3:** Interrelation between Immunoexpression for TNF-α in the Different Tumors Stagings with Education Levels from the Patients Treated at Haroldo Juaçaba Hospital (Ceará Cancer Institute).

Stage	↓ TNF-α			↑ TNF-α		
	I/II	III/IV	p-Valor	I/II	III/IV	p-Valor
Education Level						
Illiterate/Incomplete	2	13	1	3	21*	0.033
Primary School	40.00%	46.40%		27.30%	67.70%	
Completed primary school/High school/	3	15		8*	10	
Higher education	60.00%	53.60%		72.70%	32.30%	

*p<0.05. x^2^ or fisher exact test; Data expressed as absolute frequency and percentage

**Table 4:** Interrelation between Immunoexpression for TNF-α in the Different Education Levels with Clinical Pathological and Socio-Demographic Variables of Patients with SCC from the Patients Treated at Haroldo Juaçaba Hospital (Ceará Cancer Institute).

	↑ TNF-α e ↓ Education Level		Other Groups		p-Valor
Clinical Pathological Variables					
Sex					
Male	15	60.00%	35	68.60%	0.456
Females	10	40.00%	16	31.40%	
Idade					
Up to 60 years	11	44.00%	32	62.70%	0.121
Over 60 years	14	56.00%	19	37.30%	
Localization					
Floor of mouth	15	60.00%	20	39.20%	0.152
Thongue	5	20.00%	21	41.20%	
Others	5	20.00%	10	19.60%	
T					
T1/2	6	24.00%	23	45.10%	0.075
T3/4	19	76.00%	28	54.90%	
N					
N0/1	16	64.00%	34	66.70%	0.818
N2/3	9	36.00%	17	33.30%	
M					
M0	24	96.00%	50	98.00%	1
M1	1	4.00%	1	2.00%	
Stage					
1 and 2	3	12.00%	13	25.50%	0.237
3 and 4	22	88.00%	38	74.50%	
Histological Gradation					
Moderately differentiated	24	96.00%	47	92.20%	0.525
Poorly differentiated	1	4.00%	4	7.80%	
Treatment					
Surgery	5	19.2%	4	8.5%	0.026
Surgery + RT	6	23.1%	26*	55.3%	
Surgery + RT + QT	15*	57.7%	17	36.2%	
Sociodemographic Factors					
Education Level					
Illiterate/incomplete primary school	25	100.0%	48	94.1%	0.216
Completed primary school/High school/Higher education	0	0.0%	3	5.9%	
Convenat					
Plubic Health System	19	76.0%	42	82.4%	0.513
Private Health System	6	24.0%	9	17.6%	
Race					
Mixed	10	40.0%	15	29.4%	0.356
No-Mixed	15	60.0%	36	70.6%	
Origin					
Metropolitan Region	16	64.0%	39	76.5%	0.253
Countryside	9	36.0%	12	23.5%	
Marital Status					
With marriage bond	10	40.0%	20	39.2%	0.507
Without marriage bond	5	20.0%	16	31.4%	
Habits	10	40.0%	15	29.4%	

*p<0.05. x^2^ or fisher exact test; Data expressed as absolute frequency and percentage

## Discussion

This study evaluate the immunostaining of cytokines in OSCC, in tissue of margin of surgical resection (MSR) and metastatic lymph nodes and the role of these cytokines in patient prognosis and survival. The clinical data showed a high prevalence of men who were less than 60 years old, an unusual age in epidemiological studies (Heo et al., 2012; Odveig et al., 2014; Liu et al., 2016). As previously described, the most prevalent tumour localization was in the tongue and floor of mouth (Odveig et al., 2014; Satochi et al., 2014), the most frequent stages were III/IV (Durazzo et al., 2005) and the main risk factor was smoking (Iamaroon et al., 2004).

The inflammatory process is vital for mammals, acting as a protector against infectious agents, traumatic injuries and cancer development. The recognition of lesions made by microorganisms, trauma or disorderly tissue growth leads to the production of many inflammatory mediators, such as cytokines (Landskron et al., 2014). However, in chronic processes, a pathologically favourable microenvironment is created, promoting the development of a series of diseases and contributing to tumour progression (Iamaroon et al., 2004).

In addition to clinical-epidemiological factors, we have shown the profile of the immunoexpression of inflammatory cytokines in the tumour, perilesion and metastasis in the lymph node and have observed that, in general, the immunoexpression of these cytokines is greater in the primary tumour or in lymph node metastasis than in MSR. A similar result has been shown in the literature, although the role of these cytokines has not yet been fully elucidated (Zhang et al., 2014). 

In our study, we did not observe a change in the immunoexpression profile of inflammatory cytokines from the primary tumour to lymph node metastasis, showing that after malignization, the immunoexpression of inflammatory mediators increases in relation to MSR and remains at a similar level when there is metastasis to the lymph node. Studies have shown that proinflammatory cytokines such as IL-1 beta, TNF-alpha, and IL-6, produced by the malignant cells themselves, are associated with increased keratinocyte motility, and consequently nodal metastasis (Voiculescu, 2016).

IL-10, an anti-inflammatory cytokine, has been correlated with antitumour activity in some studies6, not only in squamous cell carcinoma but also in other tumours, such as breast and ovarian carcinomas (Ranelletti et al., 2001). In our study, IL-10 was not associated with a better prognosis, although its immunoexpression is prevalent in tumour tissue and metastasis in the lymph node, as previously described (Arantes et al., 2016).

In addition to the characterization of cytokines, infiltration of immunological cells has been used to evaluate the prognosis of malignancies (Fang et al., 2017). Our study shows CD68-positive cells, a marker that has been used as a pan-macrophage marker (Arun et al. 2009), to be more present in primary tumour and metastasis in lymph nodes than in MSR, as previously described (Madrek et al., 2012). Although this is not associated with better or worse prognosis, a similar result was found in the study by Fang et al., (2017). Perhaps phenotypic macrophage differentiation, M1 or M2, is necessary to better evaluate the role of cytokines in the progression of squamous cell carcinoma (Alvez, 2018).

These clinical data are complemented by a new approach. We have shown that the immune profile of some pro-inflammatory cytokines of innate response is increased in the tumour or lymph node metastasis, compared to MSR squamous epithelium, which was also demonstrated (Balkwill and Mantovani, 2001). Despite the role of these mediators remaining unclear, the augmentation of expression of inflammatory markers has been shown in the literature (Zang, 2014). Balkwill and Mantovani (2001) demonstrated that inflammatory cells and cytokines in peritumour stroma contribute more to the progression and development of the tumour than to the host immune response and that macrophage activation can lead to more DNA damage, anti-neoplastic gene suppression, angiogenesis and local destruction (Landskron et al., 2014).

In support of these data, in our study, the patients with high TNF-α demonstrated lower survival. TNF-α is involved in both carcinogenesis and tumour progression, assisting in angiogenesis and tumour invasion (Eirón et al., 2012). In cell culture of OSCC, TNF-α reduces cell viability (Ni et al., 2015), and many chemotherapies lead to cell death by activation of TNF-α receptors mediated by Bcl-2 and caspase-independent pathways (Chan et al., 2012). TNF-α receptor activation can activate cell death leading to apoptosis, NF-kB induction, and overproduction of IL-1β, IL-6 and other chemokines (Popa et al., 2007). 

However, this inflammatory microenvironment leads to macrophage migration and production of oxygen-reactive species, leading to metastasis in lymph nodes (León et al., 2015). TNF-α autocrine activation is associated with tumour growth (Landskron et al., 2014) through mechanisms such as increased cell proliferation that are dependent on NF-κB activation (Tang et al., 2017); reduction of apoptosis and inactivation tumour suppressor genes (Zhang et al., 2014); increased synthesis of matrix metalloproteinases, which is closely related to the local invasion and the distance of malignant cells (Tang et al., 2017); induction of metastasis and angiogenesis (Zhou et al., 2015); and increased expression in endothelial cells of proteins that mediate the adhesion of VCAM-1 (Song et al., 2012). 

This tumour growth results in a greater tumour staging that is linked to worse prognosis and higher mortality (Iamaroon et al., 2004). A previous study conducted by this group of researchers (Dantas et al., 2016) also observed that schooling directly influences patient survival. Oral cancer is related to low education, which may be partly due to poor access to disease information in general, including the diagnosis and treatment of oral cancer (Atununes et al., 2001).

It was also observed, similar to the present study, that patients with low education and high immunoexpression for TNF-α showed a worse survival rate, in addition to greater staging and use of surgery associated with radiotherapy and chemotherapy as a treatment of choice (Krishnan et al., 2014). In previous studies, patients with stage III and IV tumours expressed more TNF-α; in the researchers’ country, patients with advanced disease stage are patients with a low level of education (Ferreira et al., 2012; Dantas et al., 2016). 

In conclusion, this work shows that there is an increased immunoexpression of inflammatory cytokines and macrophages in the primary tumour in relation to the MSR, but without any major changes in metastasis to the lymph nodes. We have also shown that TNF-α, as the only inflammatory cytokine that directly influences the survival of patients with oral cancer, should be considered for further studies, possibly proving to be a predictor of prognosis of squamous cell carcinoma. This study, along with further studies, may also verify the social profile behind the worst prognosis of this neoplasia. Therefore, taking into consideration that patients with a low level of schooling show lower survival, this study shows that there is a fundamental need for public health policy intervention in populations with this profile. In terms of the limitations of this work, we cannot demonstrate by which mechanisms TNF-α acts on the progression of the tumour and the consequent increase in the mortality of these patients.
